# Supplementary material for: Microgliopathy as a primary mediator of neuronal death in models of Friedreich’s Ataxia
Source: Nat Commun. 2025 Nov 29;17:81. doi: 10.1038/s41467-025-66710-y (PMC12770375; doi:10.1038/s41467-025-66710-y)
Supplement: Supplementary file 1 — Supplementary Information [file 41467_2025_66710_MOESM1_ESM.pdf]

Microgliopathy as a primary mediator of neuronal death in models of Friedreich's Ataxia

Carla Pernaci[1,2], Avalon Johnson[1,2], Sydney Gillette[1,2], Anna S. Warden[1,2], Chad McCormick[1,2], Sammy Weiser-Novak[3], Gabriela Ramirez[1,2], Emily H. Broersma[1,2], Priyanka Mishra[4], Anusha Sivakumar[4], Stephanie Cherqui[4†], Nicole G. Coufal[1,2†*]

[1] Department of Pediatrics, University of California, San Diego, La Jolla, CA 92093, US
[2] Sanford Consortium for Regenerative Medicine, La Jolla, CA 92037, USA
[3] Salk Institute for Biological Studies, La Jolla, CA 92037, USA
[4] Department of Pediatrics, Division of Genetics, University of California, San Diego, San Diego, CA, United States

† Contributed equally

*Corresponding author

Email corresponding author: ncoufal@health.ucsd.edu

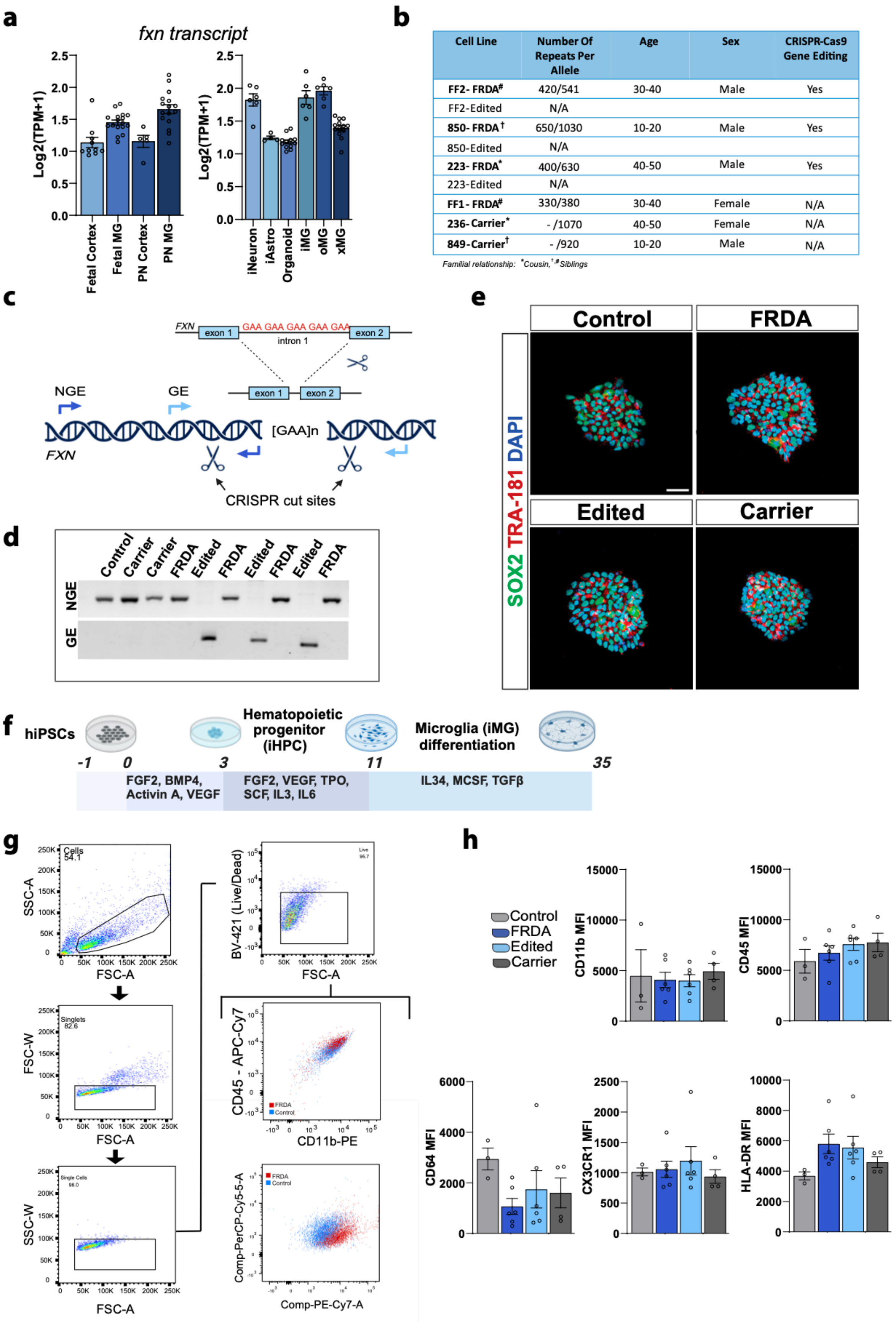
a
fxn transcript
Log2(TPM+1)
Fetal Cortex
Fetal MG
PN Cortex
PN MG
iNeuron
iAstro
Organoid
iMG
oMG
xMG
b
Cell Line | Number Of Repeats Per Allele | Age | Sex | CRISPR-Cas9 Gene Editing
FF2- FRDA# | 420/541 | 30-40 | Male | Yes
FF2-Edited | N/A
850- FRDA† | 650/1030 | 10-20 | Male | Yes
850-Edited | N/A
223- FRDA* | 400/630 | 40-50 | Male | Yes
223-Edited | N/A
FF1- FRDA# | 330/380 | 30-40 | Female | N/A
236- Carrier* | - /1070 | 40-50 | Female | N/A
849-Carrier† | - /920 | 10-20 | Male | N/A
Familial relationship: *Cousin, †,#Siblings
c
FXN
exon 1
GAA GAA GAA GAA GAA
intron 1
exon 2
NGE
GE
[GAA]n
CRISPR cut sites
d
Control
Carrier
Carrier
FRDA
Edited
FRDA
Edited
FRDA
Edited
FRDA
NGE
GE
e
Control
FRDA
Edited
Carrier
SOX2 TRA-181 DAPI
f
hiPSCs
Hematopoietic progenitor (iHPC)
Microglia (iMG) differentiation
-1
0
3
11
35
FGF2, BMP4, Activin A, VEGF
FGF2, VEGF, TPO, SCF, IL3, IL6
IL34, MCSF, TGFβ
g
Cells 54.1
SSC-A
FSC-A
Singlets 82.6
FSC-W
Single Cells 98.0
SSC-W
BV-421 (Live/Dead)
Live 95.7
CD45 - APC-Cy7
CD11b-PE
FRDA
Control
Comp-PerCP-Cy5-5-A
Comp-PE-Cy7-A
h
Control
FRDA
Edited
Carrier
CD11b MFI
CD45 MFI
CD64 MFI
CX3CR1 MFI
HLA-DR MFI

Supplementary Figure 1. Generation of FRDA and gene-edited isogenic CRISPR/Cas9 targeted iPSCs. a) *Fxn* transcript levels in healthy donors from a published dataset[19]. Left: *Fxn* expression in primary fetal and post-natal microglia and whole cortex. Right: *Fxn* expression in iPSC derived cell types in induced (i) neurons, astrocytes, organoid and microglia, xenotransplanted microglia (xMG) and organoid cocultured microglia (oMG). (TPM = transcripts per million). **b)** List of patient cohorts used in this study. Cell line and patient characteristics are listed, including GAA repeat numbers, the gene editing approach used, age, sex, and familial relationship, FRDA, control and gene edited iPSCs have been reported previously [8], carriers are novel in this study. **c)** Schematic overview of the dual CRISPR guide approach *Created in BioRender. Coufal, N. (2025) https://BioRender.com/m5qd597*. **d)** Gel electrophoresis of PCR products from the gene-edited (GE) (401 bp) and non-gene-edited (NGE) (313 bp) primers confirming the success of our CRISPR/Cas9 approach. **e)** Confocal images of pluripotency markers, SOX2 and TRA-181 in iPSCs cell lines. (Scale bars 50 mm). **f)** Schematic overview of microglia differentiation protocol created *in BioRender. Coufal, N. (2025) https://BioRender.com/d4rnu8b* **g)** Mean fluorescence intensity (MFI) of microglia markers including CD45, CD11b, CX3CR1, and CD64 by flow cytometry. **h)** Representative flow cytometry data of control and FRDA microglia for markers.

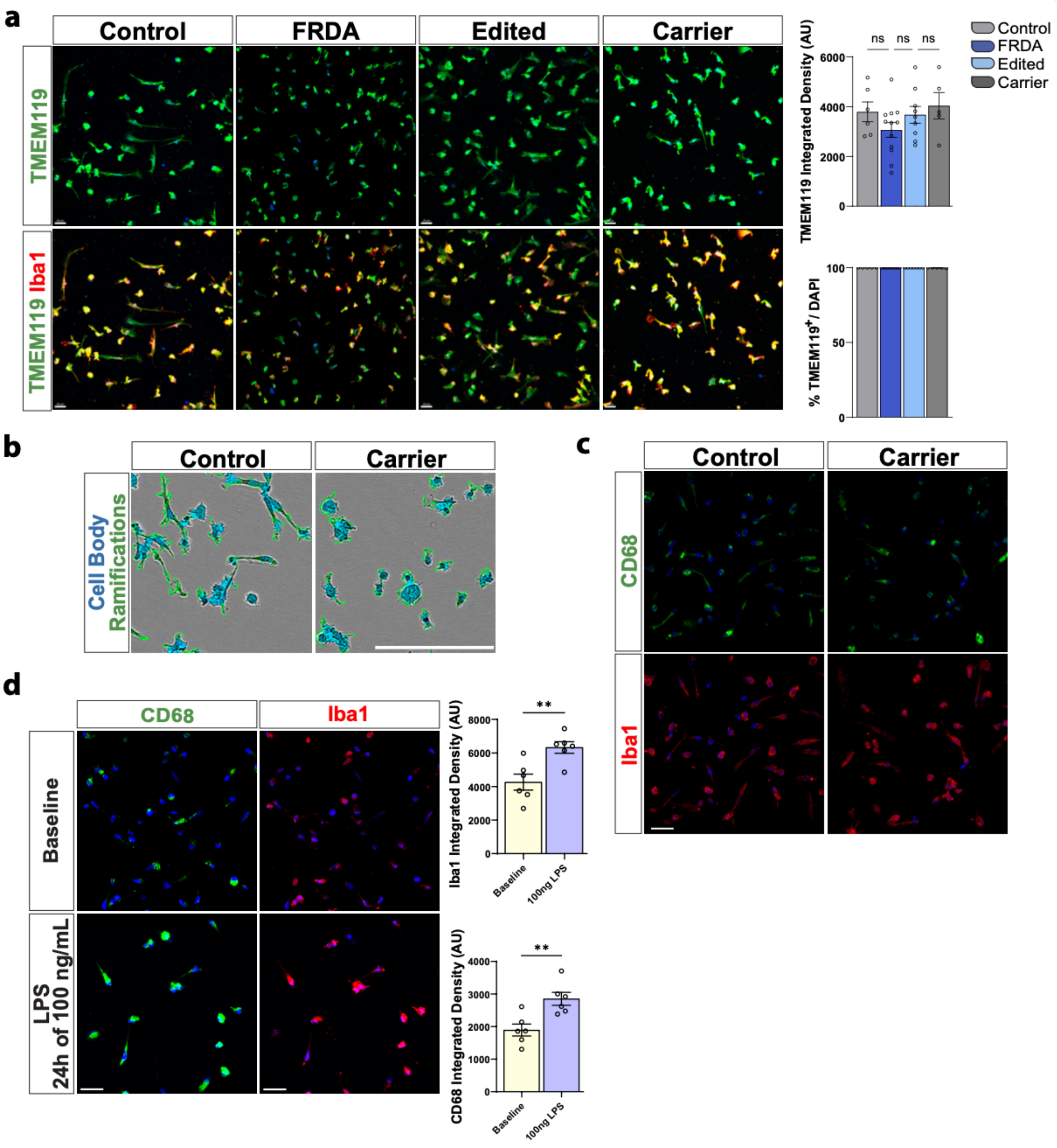

a
Control
FRDA
Edited
Carrier
TMEM119
TMEM119 Iba1
TMEM119 Integrated Density (AU)
ns
ns
ns
6000
4000
2000
0
Control
FRDA
Edited
Carrier
% TMEM119+/ DAPI
100
50
0
b
Control
Carrier
Cell Body
Ramifications
c
Control
Carrier
CD68
Iba1
d
CD68
Iba1
Baseline
LPS
24h of 100 ng/mL
Iba1 Integrated Density (AU)
8000
6000
4000
2000
0
**
Baseline
100ng LPS
CD68 Integrated Density (AU)
4000
3000
2000
1000
0
**
Baseline
100ng LPS

Supplementary Figure 2. Generation and characterization of iPSCs derived microglia cells a) Confocal images of transmembrane protein 119 (TMEM119) as mature microglia marker (left). Quantification of TMEM119 integrated density per cell and percentage of $TMEM119^+$ cells/DAPI showing the efficacy of the microglia differentiation protocol (right). **b)** Representative images for the high-content image analysis of iMG ramification length and branch points **c)** Representative confocal images for CD68 and Iba1 expression in healthy controls and carriers. **d)** Confocal images of CD68 and Iba1 expression at baseline and after 100ng/mL of LPS stimulation for 24 h in two healthy control donor lines. Two controls, four FRDA, three gene-edited and two familial carrier lines for panel a-c and two healthy controls in panel e. Each dot in the plot represents a biological replicate with N = 3 independent experiments / line. Integrated Density (AU: arbitrary units) has been quantified per each cell, with at least 80 iMG cells/line/experiment analyzed. Data are presented as Mean ±SEM and analyzed by one-way ANOVA with Tukey's post-hoc testing. *$p < 0.05$, **$p < 0.01$, ***$p < 0.001$, ****$p < 0.0001$. Scale bars c) 20 μm, d) 30 μm, e) 30 μm.

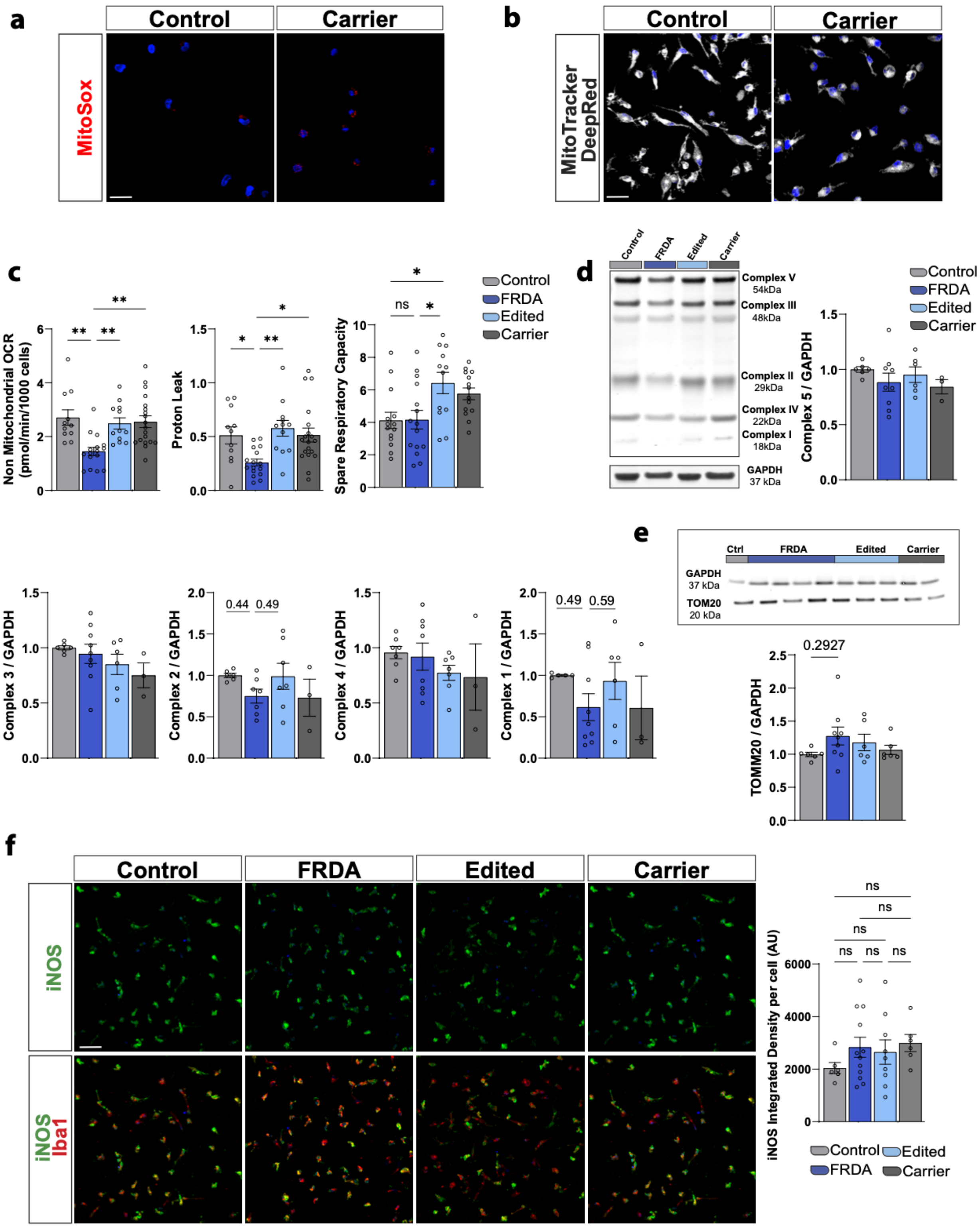
a
Control
Carrier
MitoSox
b
Control
Carrier
MitoTracker DeepRed
c
Control
FRDA
Edited
Carrier
Non Mitochondrial OCR (pmol/min/1000 cells)
Proton Leak
Spare Respiratory Capacity
ns
d
Control
FRDA
Edited
Carrier
Complex V 54kDa
Complex III 48kDa
Complex II 29kDa
Complex IV 22kDa
Complex I 18kDa
GAPDH 37 kDa
Complex 5 / GAPDH
e
Ctrl
FRDA
Edited
Carrier
GAPDH 37 kDa
TOM20 20 kDa
0.2927
TOMM20 / GAPDH
Complex 3 / GAPDH
Complex 2 / GAPDH
0.44
0.49
Complex 4 / GAPDH
Complex 1 / GAPDH
0.49
0.59
f
Control
FRDA
Edited
Carrier
iNOS
iNOS Iba1
iNOS Integrated Density per cell (AU)
ns

Supplementary Figure 3. Mitochondrial characterization of iMGs. a) Representative confocal images for MitoSox expression in healthy controls and carriers. **b)** Representative confocal images for MitoTracker deep red expression in healthy controls and carriers. **c)** Non-mitochondrial oxygen consumption and proton leak in iMGs from the Seahorse Mito Stress test. **d)** Representative western blot (WB) measuring the expression levels of the mitochondria electron transport chain (ETC) complexes and its quantification normalized over GAPDH. **e)** Representative western blot (WB) measuring the expression levels of the mitochondria membrane protein TOMM20 and its quantification normalized over GAPDH. **f)** Confocal images of iNOS expression in mature microglia and its quantification. Two controls, three FRDA, two gene-edited and two familial carrier lines/each for a-e and two controls, four FRDA, three gene-edited and two familial carrier lines for panel f. Data are presented as mean ±SEM and analyzed by one-way ANOVA with Tukey's post-hoc testing. $^{*}p < 0.05$, $^{**}p < 0.01$, $^{***}p < 0.001$, $^{****}p < 0.0001$. Each dot in the plot represents a biological replicate with N = 3 independent experiments / line. Integrated Density (AU: arbitrary units) has been quantified per each cell, with at least 80 iMG cells/line/experiment analyzed. Scale bars a) 30 μm, b) 30 μm, f) 50 μm.

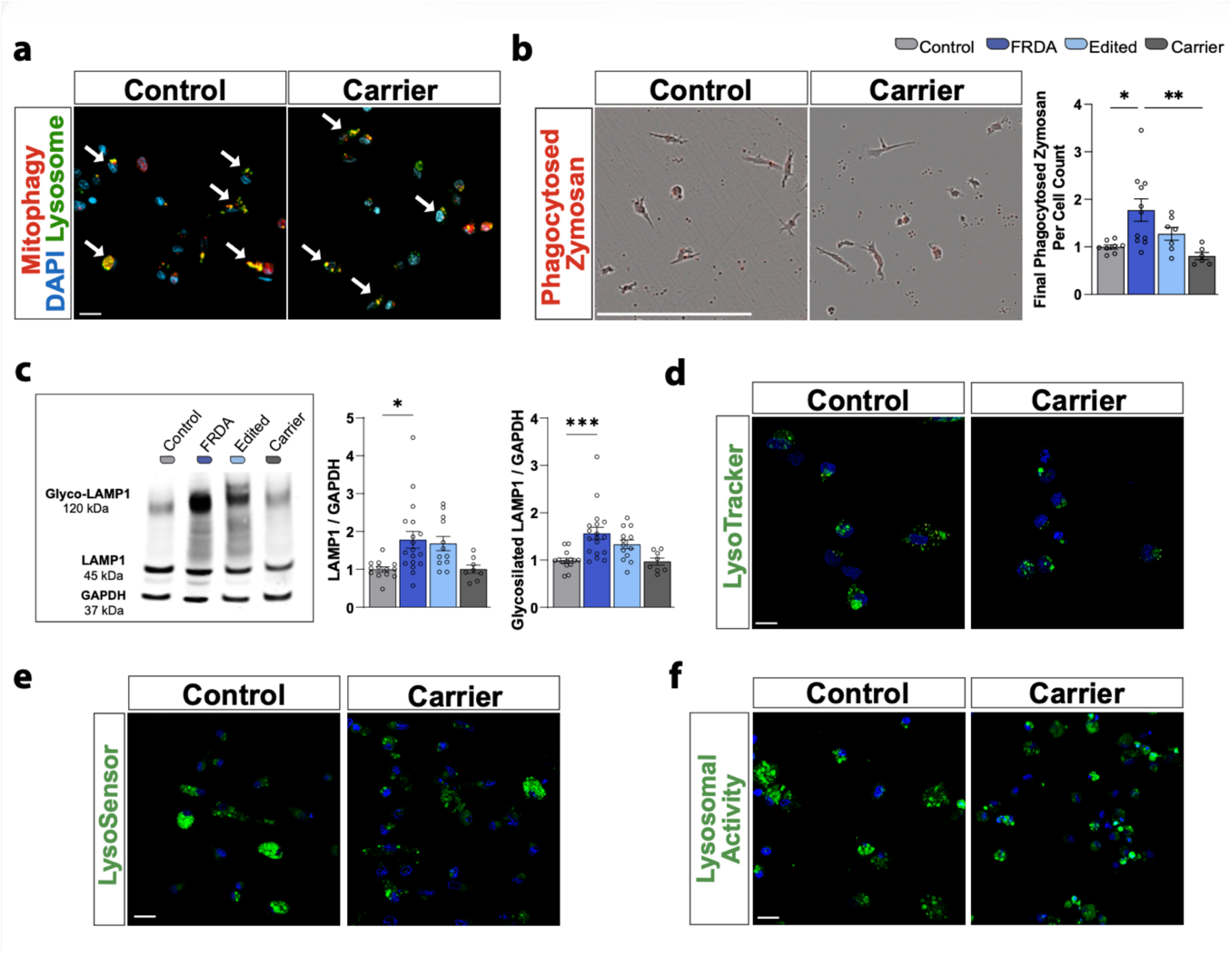

Supplementary Figure 4. Lysosomal characterization in iMGs.a) Representative confocal images for mitophagy expression in healthy controls and carriers. **b)** Representative images for zymosan phagocytosis in healthy controls and carriers. Scale bar, 200 µm. **c)** Representative western blot for LAMP1 (left). Quantitation of LAMP1 and glycosylated LAMP1 levels normalized to GAPDH (right). **d)** Representative confocal images for LysoTracker expression in healthy controls and carriers. **e)** Representative confocal images for LysoSensor expression in healthy controls and carriers. **f)** Representative confocal images for Lysosomal Activity assay expression in healthy controls and carriers. Data are presented as mean ±SEM and analyzed by two-way ANOVA with Tukey's correction where *$p < 0.05$, **$p < 0.01$, ***$p < 0.001$, ****$p < 0.0001$. Scale bars a) 20 µm, b) 200 µm, d) 15 µm, e) 30 µm, f) 30 µm.

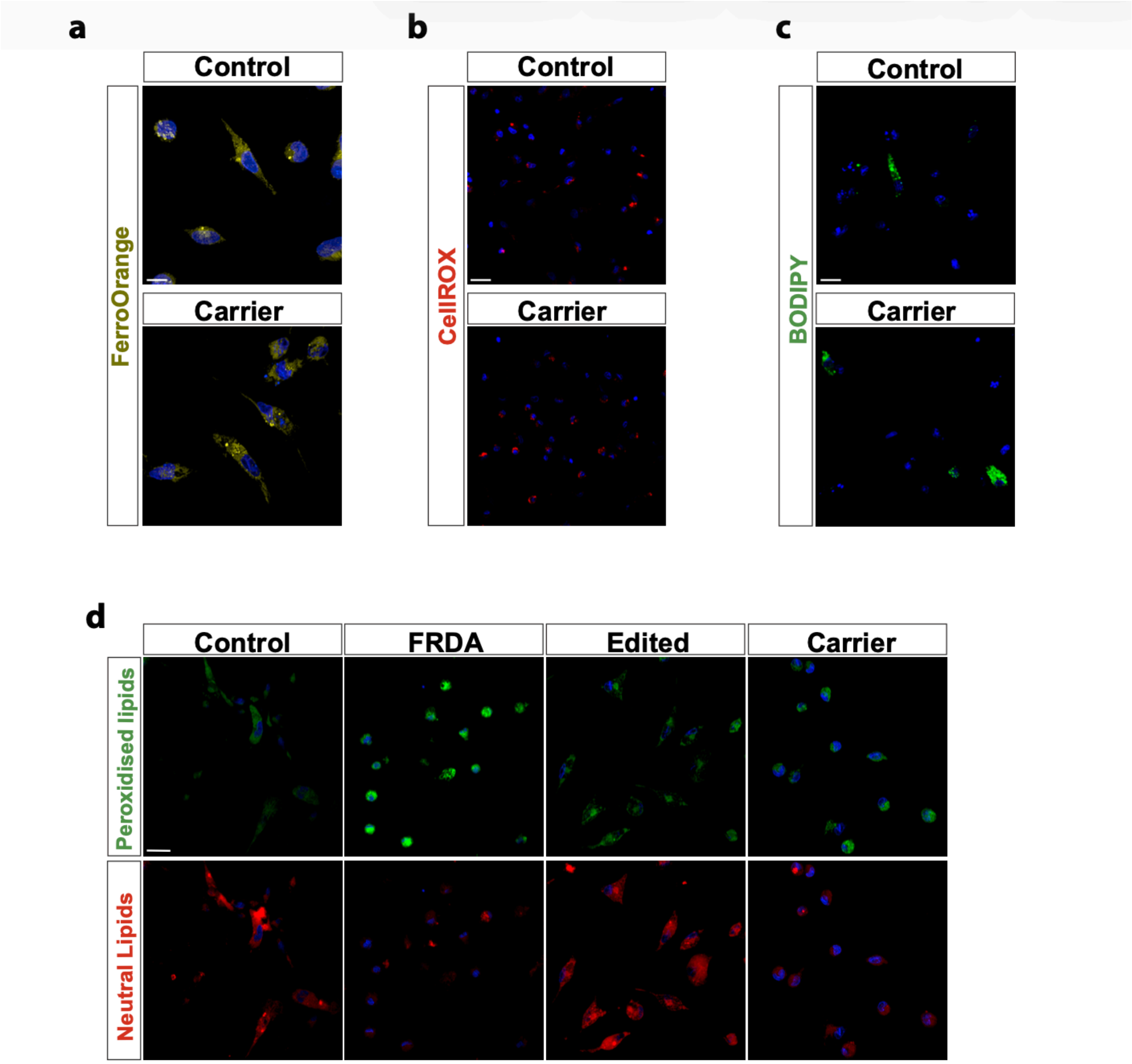

Supplementary Figure 5. Cellular homeostasis in mature microglia a) Representative confocal images for FerroOrange expression in healthy controls and carriers. **b)** Representative confocal images for CellROX expression in healthy controls and carriers. c**)** Representative confocal images for BODIPY expression in healthy controls and carriers. **f)** Representative confocal images for Lipid peroxidation. Scale bars a) 10 µm, b) 30 µm, c) 20 µm, d) 20 µm.

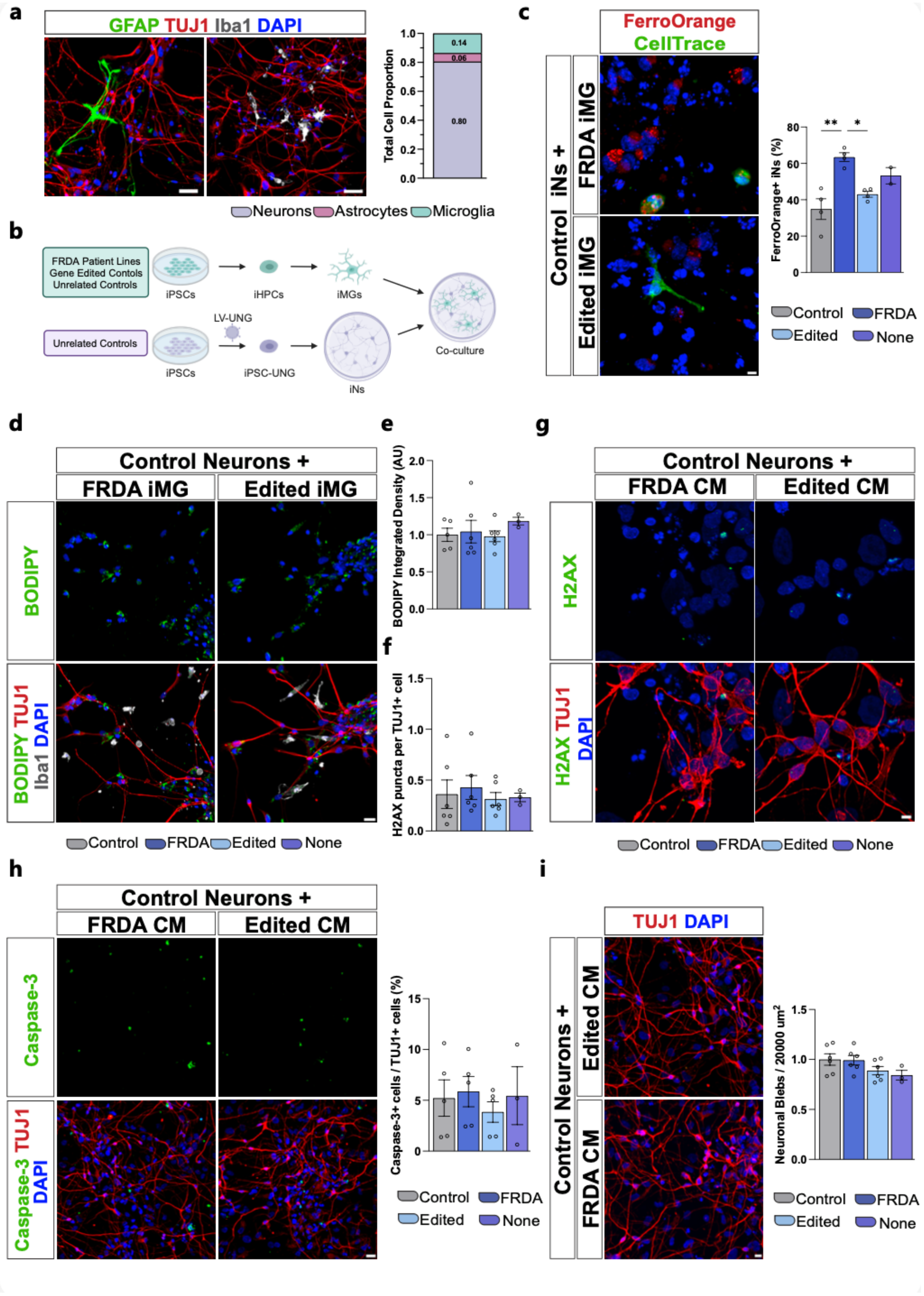

a
GFAP TUJ1 Iba1 DAPI
Total Cell Proportion
0.14
0.06
0.80
Neurons Astrocytes Microglia
b
FRDA Patient Lines
Gene Edited Controls
Unrelated Controls
iPSCs
iHPCs
iMGs
Unrelated Controls
LV-UNG
iPSC-UNG
iNs
Co-culture
c
FerroOrange
CellTrace
Control iNs +
FRDA iMG
Edited iMG
FerroOrange+ iNs (%)
Control FRDA
Edited None
d
Control Neurons +
FRDA iMG
Edited iMG
BODIPY
BODIPY TUJ1 Iba1 DAPI
Control FRDA Edited None
e
BODIPY Integrated Density (AU)
f
H2AX puncta per TUJ1+ cell
g
Control Neurons +
FRDA CM
Edited CM
H2AX
H2AX TUJ1 DAPI
Control FRDA Edited None
h
Control Neurons +
FRDA CM
Edited CM
Caspase-3
Caspase-3 TUJ1 DAPI
Caspase-3+ cells / TUJ1+ cells (%)
Control FRDA
Edited None
i
TUJ1 DAPI
Control Neurons +
Edited CM
FRDA CM
Neuronal Blebs / 20000 um²
Control FRDA
Edited None

Supplementary Figure 6. Characterization of healthy neurons co-cultured with iMG or treated with iMG conditioned media. a) Representative images (left) and quantification (right) of cell type proportions present in co-culture model. Staining includes TUJ1 for neurons, GFAP for astrocytes and IBA1 for microglia. **b)** Schematic of induced neuron (iN) and microglia co-culture created in *BioRender. Coufal, N. (2025)* https://BioRender.com/2qdya74 **c)** FerroOrange staining for intracellular iron (left) and quantitation (right) in healthy iNs co-cultured with control, FRDA or gene-edited iMG. iMG labeled with CellTrace CFSE dye (green) prior to co-culture. **d-e)** Immunostaining (left) and quantification (right) of neutral lipids (BODIPY) in healthy neurons co-cultured with iMG (AU: arbitrary units). **f-g)** Immunostaining (right) and quantification (left) of γH2AX for foci of DNA damage in healthy $TUJ1^+$ neurons cultured with iMG-conditioned media. **h)** Immunostaining (left) and quantification (right) for cleaved caspase-3 in healthy $TUJ1^+$ neurons cultured with iMG-conditioned media. **i)** Immunostaining for TUJ1 (left) and quantification (right) of neuronal blebbing in healthy neurons cultured with iMG-conditioned media. Data normalized to control. n = 2 FRDA, 2 edited, and 2 non-related controls with 3 biologically independent replicates for each iMG cell line. Data is represented as mean ±SEM and analyzed with one-way ANOVA with Tukey's posthoc testing where $^*p < 0.05$, $^{**}p < 0.01$, $^{***}p < 0.001$, $^{****}p < 0.0001$. Scale bars a) 40 μm, c) 5 μm, d) 20 μm, g) 5 μm, h) 20 μm, i) 10 μm.

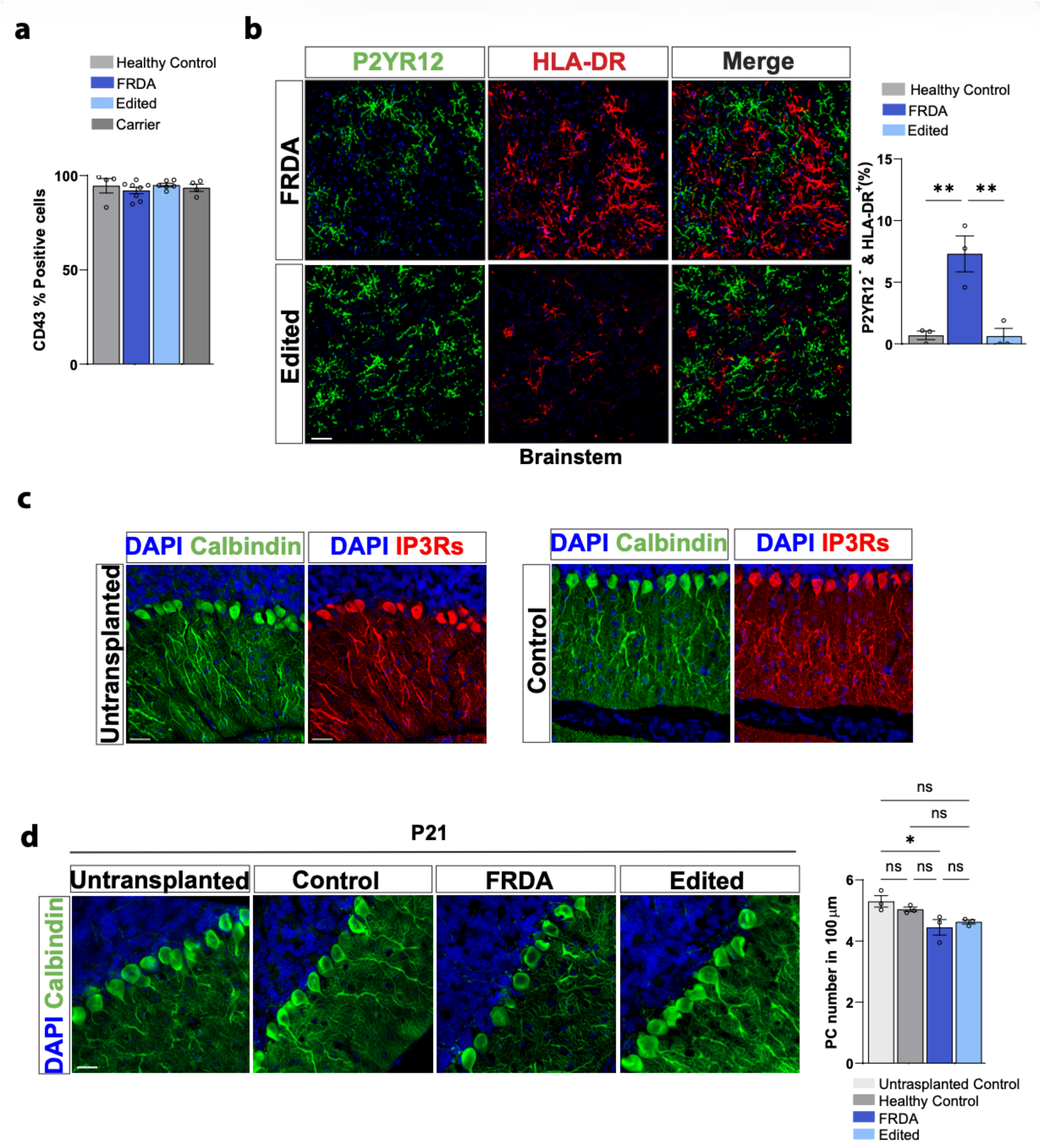

Supplementary Figure 7. iHPCs xenotransplantation a) Percentage of CD43^{+} iHPCs cells assessed by flow cytometry. **b)** Representative confocal images of the homeostatic marker P2YR12 and the activated microglia marker HLA-DR in the brain stem of adult mice. **c)** Representative confocal images for Calbindin and IP3Rs used to assess Purkinje cell number in untransplanted control mice and in FIRE mice xenotransplanted with healthy donor iHPCs. **d)**

Representative confocal images at postnatal day 21 for Calbindin. Quantification of Purkinje cells in juvenile mice (right). Data is represented as mean ±SEM and analyzed with one-way ANOVA with Tukey's posthoc testing where $*p < 0.05$, $**p < 0.01$, $***p < 0.001$, $****p < 0.0001$. Scale bars b) 50 μm, c-d) 30 μm.

Supplementary Table 1: Antibodies

Antibody	Manufacturer	Catalog #	Dilution	Application
Goat anti-Iba1	Abcam	ab5076	1:500	IF
Rabbit anti-P2RY12	Atlas	HPA014518	1:500	IF
Rabbit anti-Frataxin	Proteintech	14147-1-AP	1:200	WB
Mouse anti-Frataxin	Abcam	ab110328	1:500	IF
Mouse anti-CD68	Dako	M0814	1:200	IF
Rabbit anti-TMEM119	Invitrogen	PA5-119902	1:50	IF
Rabbit anti-TOM20	Proteintech	11802-1-AP	1:500/ 1:5000	IF/WB
Rabbit anti-Sox2	Cell Signaling Technology	2748S	1:250	IF
Mouse anti-Tra-181	EMD Mill	4381	1:500	IF
Rabbit anti-Ku80	Abcam	ab80592	1:100	IF

Rabbit anti-iNOS	Invitrogen	PA1-036	1:200	IF
Total OXPHOS Human WB Antibody Cocktail	Abcam	ab110411	1:1000	WB
Mouse anti-GAPDH	Cell Signaling	2118	1:10,000	WB
Rabbit anti-beta actin	GeneTex	GTX110564	1:10,000	WB
Rabbit anti-LAMP1	abcam	ab24170	1:1000	WB
Rabbit anti-H2AX	Cell Signaling	9718	1:200	IF
Mouse anti-TUJ1	Biolegend	86298	1:500	IF
Rabbit anti-TUJ1	Biolgened	802001	1:1000	IF
Chicken anti-GFAP	Millipore Sigma	AB5541	1:1000	IF
Rabbit anti-cleaved Caspase-3	Cell Signaling	9661	1:400	IF
Mouse anti-8-OHdG	Santa Cruz Biotechnology	sc-393871	1:200	IF
Rabbit anti-AXL	Cell Signaling	8661S	1:500	WB
Rabbit anti-Calbindin	Abcam	ab108404	1:1000	IF
Rabbit anti-HLA DR	Abcam	ab92511	1:1000	IF

Chicken anti-HSP60	EnCor Biotechnology	CPCA-HSP60	1:500	IF
Mouse anti-NeuN	Abcam	ab104224	1:200	IF
Rabbit anti-IP3R-I	Invitrogen	PA1-901	1:200	IF
Donkey anti-goat, Alexa Fluor 555	Invitrogen	A21432	1:1000	IF
Donkey anti-goat, Alexa Fluor 488	Invitrogen	ab150129	1:1000	IF
Donkey anti-goat, Alexa Fluor 647	Invitrogen	A21447	1:1000	IF
Donkey anti-rabbit, Alexa Fluor 488	Invitrogen	A32790	1:1000	IF
Donkey anti-rabbit, Alexa Fluor 555	Invitrogen	A31572	1:1000	IF
Donkey anti-rabbit, Alexa Fluor 647	Invitrogen	A31573	1:1000	IF
Donkey anti-mouse, Alexa Fluor 488	Invitrogen	A21202	1:1000	IF
Donkey anti-mouse, Alexa Fluor 555	Invitrogen	A31570	1:1000	IF
Donkey anti-mouse, Alexa Fluor 647	Invitrogen	A31571	1:1000	IF

Donkey anti-chicken, Alexa Fluor 488	Invitrogen	A78948	1:1000	IF
Donkey anti-chicken, Alexa Fluor 568	Invitrogen	A78950	1:1000	IF
Donkey anti-chicken, Alexa Fluor 647	Invitrogen	A78952	1:1000	IF
Goat anti-Mouse, Alexa Fluor Plus 800	Invitrogen	A32730	1:10,000	WB
Goat anti-Rabbit, Alexa Fluor Plus 680	Invitrogen	A32734	1:10,000	WB
Horse anti-mouse, HRP-linked Antibody	Cell Signaling Technology	7076S	1:5000	WB
Hoescht	Thermo Scientific	62249	20mM	IF
DAPI	Thermo Scientific	62248	1:1000	IF
CX3CR1	Biolegend	341614	1:100	FACS
CD68	Biolegend	333812	1:100	FACS
CD11b	Biolegend	301306	1:100	FACS
CD45	Biolegend	304014	1:100	FACS
CD43	Biolegend	143207	1:100	FACS

APC	Biolegend	400122	1:300	FACS
PerCP-Cy5.5	Biolegend	400632	1:300	FACS
488 FITC	Biolegend	400129	1:300	FACS
PE	Biolegend	400112	1:300	FACS
PE-Cy7	Biolegend	400232	1:300	FACS
APC Cy7	Biolegend	400128	1:300	FACS
Zombie Violet	Biolegend	423114	1:1000	FACS

Supplementary Table 2: Reagents

Reagent	Manufacturer	Catalog #
MitoSox Red	Invitrogen	M36008
MitoTracker Green FM	Cell Signaling Technologies	9074
MitoTracker Deep Red	Invitrogen	M22426
Mitophagy Detection Kit	Dojindo	MD01-10
Seahorse XF Cell Mito Stress Test Kit	Agilenz	103015-100
LysoTracker	Invitrogen	L7528

LysoSensor Green	Invitrogen	L7535
Lysosome Activity Assay	Abcam	ab234622
pHrodo Zymosan beads	Invitrogen	35364
Ferro Orange	Dojindo	F374-10
Cell ROX Orange Reagent	Invitrogen	C10443
pHrodo Red, SE	Invitrogen	P36600
BODIPY 493/503	Invitrogen	D3922
CellTrace Violet	Invitrogen	C34564
CellTrace Green CMFDA	Invitrogen	C2925
CellTrace Far Red	Invitrogen	C34564
Image-iT Lipid Peroxidation Kit	Thermo Scientific	C10445
Lipopolysaccharide (LPS) Solution	Invitrogen	00-4976-03
