# Supplementary material for: Microgliopathy as a primary mediator of neuronal death in models of Friedreich’s Ataxia
Source: Nat Commun. 2025 Nov 29;17:81. doi: 10.1038/s41467-025-66710-y (PMC12770375; doi:10.1038/s41467-025-66710-y)
Supplement: Supplementary file 2 — Reporting Summary [file 41467_2025_66710_MOESM2_ESM.pdf]

nature portfolio

Corresponding author(s): Nicole Coufal

Last updated by author(s): 10/31/2025

Reporting Summary

Nature Portfolio wishes to improve the reproducibility of the work that we publish. This form provides structure for consistency and transparency in reporting. For further information on Nature Portfolio policies, see our Editorial Policies and the Editorial Policy Checklist.

Please do not complete any field with "not applicable" or n/a. Refer to the help text for what text to use if an item is not relevant to your study.
For final submission: please carefully check your responses for accuracy; you will not be able to make changes later.

Statistics

For all statistical analyses, confirm that the following items are present in the figure legend, table legend, main text, or Methods section.

n/a	Confirmed	
☐	☒	The exact sample size (*n*) for each experimental group/condition, given as a discrete number and unit of measurement
☐	☒	A statement on whether measurements were taken from distinct samples or whether the same sample was measured repeatedly
☐	☒	The statistical test(s) used AND whether they are one- or two-sided<br>*Only common tests should be described solely by name; describe more complex techniques in the Methods section.*
☐	☒	A description of all covariates tested
☐	☒	A description of any assumptions or corrections, such as tests of normality and adjustment for multiple comparisons
☐	☒	A full description of the statistical parameters including central tendency (e.g. means) or other basic estimates (e.g. regression coefficient) AND variation (e.g. standard deviation) or associated estimates of uncertainty (e.g. confidence intervals)
☐	☒	For null hypothesis testing, the test statistic (e.g. *F*, *t*, *r*) with confidence intervals, effect sizes, degrees of freedom and *P* value noted<br>*Give P values as exact values whenever suitable.*
☒	☐	For Bayesian analysis, information on the choice of priors and Markov chain Monte Carlo settings
☒	☐	For hierarchical and complex designs, identification of the appropriate level for tests and full reporting of outcomes
☒	☐	Estimates of effect sizes (e.g. Cohen's *d*, Pearson's *r*), indicating how they were calculated

Our web collection on statistics for biologists contains articles on many of the points above.

Software and code

Policy information about availability of computer code

Data collection	Incucyte S3 live imaging software v2022B (Sartorius Bioanalytical Instruments Inc).
Data analysis	Software: Graphpad Prism 10, IMARIS (Oxford Instrument), FlowJo v10, Fiji and ImageJ.

For manuscripts utilizing custom algorithms or software that are central to the research but not yet described in published literature, software must be made available to editors and reviewers. We strongly encourage code deposition in a community repository (e.g. GitHub). See the Nature Portfolio guidelines for submitting code & software for further information.

Data

Policy information about availability of data

All manuscripts must include a data availability statement. This statement should provide the following information, where applicable:
- Accession codes, unique identifiers, or web links for publicly available datasets
- A description of any restrictions on data availability
- For clinical datasets or third party data, please ensure that the statement adheres to our policy

All relevant data are included in the paper and/or in supplementary information file. Source data file provided.

Research involving human participants, their data, or biological material

Policy information about studies with human participants or human data. See also policy information about sex, gender (identity/presentation), and sexual orientation and race, ethnicity and racism.

Reporting on sex and gender	Sex and gender from patient-derived microglia are reported in Supplementary figure1B. For postmortem human tissue characterization (Fig.1A) subjects sex are the following: FRDA two males and one female and similarly two males and one female for healthy donor lines.
Reporting on race, ethnicity, or other socially relevant groupings	Race from patient-derived microglia: all caucasian. For postmortem human tissue characterization subjects race are the following: FRDA two whites and one black or african , for healthy donor controls are two whites and one black.
Population characteristics	clinical diagnoses for all the disease patient line and post mortem human tissue: Friedreich ataxia, no clinical brain diagnoses for healthy controls.
Recruitment	LCL cell lines were obtained from Coriell, postmortem human tissue from NeuroBioBank (NIH)
Ethics oversight	Approved by the Institutional Review Board of the University of California, San Diego.

Note that full information on the approval of the study protocol must also be provided in the manuscript.

Field-specific reporting

Please select the one below that is the best fit for your research. If you are not sure, read the appropriate sections before making your selection.

☒ Life sciences ☐ Behavioural & social sciences ☐ Ecological, evolutionary & environmental sciences

For a reference copy of the document with all sections, see nature.com/documents/nr-reporting-summary-flat.pdf

Life sciences study design

All studies must disclose on these points even when the disclosure is negative.

Sample size	Four FRDA patient lines were generated, three of which underwent CRISPR/Cas9 gene editing approach to generate isogenic contro l lines together with two additional familial carriers and two unrelated healthy donor lines. Each experiment was replicated independently utilizing multiple lines/genotype. For all experiments, no statistical methods were used to pre-determine sample size but our sample sizes are similar to those reported in previous publications (Nott et al 2019, McQuade et al 2020).
Data exclusions	Data that didn't met quality control metrics were excluded.
Replication	All assays were successfully replicated 2-4 times; quantification and statistics are run on combined replicate experiments. Only findings that were independently replicated were included within the manuscript.
Randomization	No randomization was performed.
Blinding	Investigator were blinded.

Reporting for specific materials, systems and methods

We require information from authors about some types of materials, experimental systems and methods used in many studies. Here, indicate whether each material, system or method listed is relevant to your study. If you are not sure if a list item applies to your research, read the appropriate section before selecting a response.

Materials & experimental systems

n/a	Involved in the study	
☐	☒	Antibodies
☐	☒	Eukaryotic cell lines
☒	☐	Palaeontology and archaeology
☐	☒	Animals and other organisms
☒	☐	Clinical data
☒	☐	Dual use research of concern
☒	☐	Plants

Methods

n/a	Involved in the study	
☒	☐	ChIP-seq
☐	☒	Flow cytometry
☒	☐	MRI-based neuroimaging

Antibodies

Antibodies used	Please see the comprehensive table within the supplementary methods that details all antibodies and concentration with supplier and catalog number utilized within this study.
Validation	Antibodies were validated by the manufacturer.

Eukaryotic cell lines

Policy information about cell lines and Sex and Gender in Research

Cell line source(s)	Eleven cell lines were utilized in this study, the EC11 cell line (with references, extensively published) and the Coriell GM03816, GM23913, GM16223, GM22264, GM16236, GM15849, GM15850 cell line.
Authentication	All cell lines were karyotyped and verified to express pluripotency markers and to be free of mycoplasma contamination.
Mycoplasma contamination	All cell lines were regularly mycoplasma tested and were negative.
Commonly misidentified lines (See ICLAC register)	N/A

Animals and other research organisms

Policy information about studies involving animals; ARRIVE guidelines recommended for reporting animal research, and Sex and Gender in Research

Laboratory animals	Adult, 8-weeks old FIRE mice used
Wild animals	N/A
Reporting on sex	Male and female were included in this study
Field-collected samples	N/A
Ethics oversight	All animal procedures described have been approved by UCSD IACUC at an AAALAC accredited facility.

Note that full information on the approval of the study protocol must also be provided in the manuscript.

Plants

Seed stocks	N/A
Novel plant genotypes	N/A
Authentication	N/A

Flow Cytometry

Plots

Confirm that:

☒ The axis labels state the marker and fluorochrome used (e.g. CD4-FITC).

☒ The axis scales are clearly visible. Include numbers along axes only for bottom left plot of group (a 'group' is an analysis of identical markers).

☒ All plots are contour plots with outliers or pseudocolor plots.

☒ A numerical value for number of cells or percentage (with statistics) is provided.

Methodology

Sample preparation	iPSC derived microglia

Instrument	BD Influx
Software	Flow-Jo software v10
Cell population abundance	Cells were analyzed but not sorted by flow cytometry.
Gating strategy	Reported in Suppl. Fig1

☒ Tick this box to confirm that a figure exemplifying the gating strategy is provided in the Supplementary Information.

This checklist template is licensed under a Creative Commons Attribution 4.0 International License, which permits use, sharing, adaptation, distribution and reproduction in any medium or format, as long as you give appropriate credit to the original author(s) and the source, provide a link to the Creative Commons license, and indicate if changes were made. The images or other third party material in this article are included in the article's Creative Commons license, unless indicated otherwise in a credit line to the material. If material is not included in the article's Creative Commons license and your intended use is not permitted by statutory regulation or exceeds the permitted use, you will need to obtain permission directly from the copyright holder. To view a copy of this license, visit http://creativecommons.org/licenses/by/4.0/
